# A day in the life: a qualitative study of clinical decision-making and uptake of neurorehabilitation technology

**DOI:** 10.1186/s12984-021-00911-6

**Published:** 2021-07-28

**Authors:** Courtney Celian, Veronica Swanson, Maahi Shah, Caitlin Newman, Bridget Fowler-King, Sarah Gallik, Kaitlin Reilly, David J. Reinkensmeyer, James Patton, Miriam R. Rafferty

**Affiliations:** 1 Shirley Ryan AbilityLab, 355 E Erie St., Chicago, IL 60611 USA; 2 Department of Mechanical and Aerospace Engineering, Henry Samueli School of Engineering, University of California, Engineering Gateway 4200, Irvine, CA 92697 USA; 3 Department of Bioengineering, University of Illinois at Chicago, 851 S Morgan St, Chicago, IL 60607 USA; 4 Department of Anatomy and Neurobiology, UC Irvine School of Medicine, University of California, Irvine, Irvine, CA 92617 USA; 5 Department of Physical Medicine and Rehabilitation, Northwestern University Feinberg School of Medicine, 420 E Superior St, Chicago, IL 60611 USA

**Keywords:** Rehabilitation technology, Occupational therapists, Physical therapists, Inpatient rehabilitation, Neurologic rehabilitation, Implementation

## Abstract

**Background:**

Neurorehabilitation engineering faces numerous challenges to translating new technologies, but it is unclear which of these challenges are most limiting. Our aim is to improve understanding of rehabilitation therapists’ real-time decision-making processes on the use of rehabilitation technology (RT) in clinical treatment.

**Methods:**

We used a phenomenological qualitative approach, in which three OTs and two PTs employed at a major, technology-encouraging rehabilitation hospital wrote vignettes from a written prompt describing their RT use decisions during treatment sessions with nine patients (4 with stroke, 2 traumatic brain injury, 1 spinal cord injury, 1 with multiple sclerosis). We then coded the vignettes using deductive qualitative analysis from 17 constructs derived from the RT literature and the Consolidated Framework for Implementation Research (CFIR). Data were synthesized using summative content analysis.

**Results:**

Of the constructs recorded, the five most prominent are from CFIR determinants of: (i) relative advantage, (ii) personal attributes of the patients, (iii) clinician knowledge and beliefs of the device/intervention, (iv) complexity of the devices including time and setup, and (v) organizational readiness to implement. Therapists characterized candidate RT as having a relative disadvantage compared to conventional treatment due to lack of relevance to functional training. RT design also often failed to consider the multi-faceted personal attributes of the patients, including diagnoses, goals, and physical and cognitive limitations. Clinicians’ comfort with RT was increased by their previous training but was decreased by the perceived complexity of RT. Finally, therapists have limited time to gather, setup, and use RT.

**Conclusions:**

Despite decades of design work aimed at creating clinically useful RT, many lack compatibility with clinical translation needs in inpatient neurologic rehabilitation. New RT continue to impede the immediacy, versatility, and functionality of hands-on therapy mediated treatment with simple everyday objects.

## Background

Rehabilitation technology (RT) is defined by the Rehabilitation Act of 1973 as “the systematic application of technologies, engineering methodologies, or scientific principles to meet the needs of and address the barriers confronted by individuals with disabilities…” [1]. Occupational and physical therapists (OTs and PTs) use a variety of measurement and therapeutic RTs to aide in their delivery of evidence-based rehabilitation. The field of neurorehabilitation engineering faces numerous challenges with translating new RT into everyday practice at all stages of development and implementation. Successful application of therapeutic RT requires development, testing, validation, clinician uptake, and patient acceptance.

There are several benefits of incorporating RT into therapy. RT can enable therapists to achieve tasks that are difficult or impossible to do without RT, such as lifting a heavy patient or measuring physiological variables [2]. RT can enable patients to achieve a higher number of movement practice repetitions, a necessary element of neuroplasticity during recovery [3, 4]. RT can increase motivation for therapy by providing physical assistance that allows patients to attempt and complete movements [5–7] or by incorporating gaming environments and quantitative feedback [8]. Finally, it can also reduce the need for providing continuous physical assistance or supervision to a patient, which can increase productivity or can increase patient access to therapeutic training [9].

Despite the observed benefits of RT, clinicians report barriers to their practical application. Barriers can arise from multiple domains such as the patient, the clinician, or the rehabilitation context [10]. Patients themselves can reject RT in favor of conventional therapy or have cognitive deficits which inhibit their participation [4]. Clinicians question the effectiveness strength and clinical necessity of the device [4]. Within the clinical setting, devices sometimes are too large and bulky to adapt use within an organization [11]. Clinician use is also influenced by institution facilitation of use, organizational culture and intention of use [2]. Outside clinical setting barriers also exist when a device is unavailable to the patient post-discharge [10].

Research suggests that clinicians function as gatekeepers to promote the implementation of new interventions [12]. The process for adopting RT into the clinic must undergo intense scrutiny before uptake including the clinical applicability, cost–benefit analysis, and safety of the device [13]. Therefore, it is vital to determine the gaps between the theoretical benefits and the practical application of such RT that would enable clinician uptake. Several previous studies have used survey methods [10, 14] or focus groups [4] to identify these gaps, but such approaches may not fully capture the real-time, pragmatic decision making that therapists must engage in during treatment sessions. Our approach here combined implementation science methodology to help make research more generalizable. Our premise is that integrating implementation science with neurorehabilitation engineering can accelerate the future integration of novel RT.

Our purpose is to describe clinician decision-making around incorporating RT into treatment sessions to improve understanding of clinician uptake, the critical step to device implementation. To provide a window into a day-in-the-life of clinician and the decision-making during a typical treatment session, we had OTs and PTs write vignettes describing a treatment session, along with their thought processes. Then we synthesized the vignette data using an implementation science framework, the Consolidated Framework for Implementation Research (CFIR), a common implementation framework used to classify the determinants (barriers and facilitators) of successful implementation [15]. From our qualitative data analysis, we were able to pinpoint several constructs mentioned in the vignettes to highlight the hurdles encountered by therapists in treatment sessions. Presenting and synthesizing vignettes will help engineering audiences to understand the practical application of the devices they develop and how to improve the success of future RT.

## Methods

### Context

The Shirley Ryan AbilityLab flagship research rehabilitation hospital includes interdisciplinary inpatient acute rehabilitation, outpatient rehabilitation, and research infrastructure. It differs from many interdisciplinary inpatient acute rehabilitation facilities in two key ways: (1) the facility was built with planned integration of state-of-the art RT in clinical areas and (2) research infrastructure is embedded in the clinical areas. The environment strives to be conducive to RT use through greater availability and integration of clinician researchers. Within this technology-encouraging context, we ask the questions of how RT is chosen for use, or if it's chosen at all.

### Vignette development

We asked three OTs and two PTs to write vignettes sharing (1) their decision-making process related to RT use, (2) describe their comfort level with RT and (3) provide 1–2 examples of a recent clinical treatment session, focusing on their clinical reasoning behind their decision to use, or not use, RT. We provided the following vignette template:

I am a PT/OT … I am a (describe yourself—technophile, technology early-adopter, skeptic, techno-phobe, or other) and normally use technology when… The barriers to my access to technology are… A patient with this diagnosis… and characteristics… Their goals were…. I had a ____ min session, I opted to do these interventions (tools/technologies) …. because…. It took me this much time to set up… I provided these instructions… The patient responded in this way… I chose not to use tools because… It worked/did not work because…

#### Analyses

We used deductive qualitative analysis to identify codes in the provided vignettes related to barriers to RT use and knowledge translation identified in literature [10, 14, 16]. We named these barriers using the CFIR framework, which explains 39 implementation constructs across 5 domains. These constructs can be barriers or facilitators, making implementation more or less difficult, respectively [15]. The codebook (Table 1) contained 15 original CFIR constructs identified in prior research [10, 14, 16]. Two constructs were added to distinguish between the attributes, knowledge and beliefs of clinicians compared to patients.

**Table 1:** CFIR domains, definitions, and occurrences in vignettes

CFIR domain	Construct*	Definitions*	Number of clinicians (of 5) & number of vignettes (of 9) mentioning (%)	Total number of times mentioned (% of 174 total mentions)	Exemplar quotes**	
					Facilitators	Barriers
Intervention characteristics	Intervention evidence strength and quality	Stakeholders’ perceptions of the quality and validity of evidence supporting the belief that the intervention will have desired outcomes	2/5 (40%) 3/9 (33.3%)	4 (2.3%)	“[the device allows delivery of] evidence-based practice regarding intensity of treatment.”	"One session would also not allow for achieving recommended frequency and duration to obtain the known benefits with use of the device"
	Cost	Costs of the intervention and costs associated with implementing the intervention including investment, supply, and opportunity costs	1/5 (20%) 1/9 (11.1%)	2 (1.1%)	[none coded]	“I would have liked to use the device … but the device was … locked in a manager's office because it is expensive” (clinician stage of change)
	Complexity	Perceived difficulty of the intervention, reflected by duration, scope, radicalness, disruptiveness, centrality, and intricacy and number of steps required to implement	5/5 (100%) 9/9 (100%)	16 (9.2%)	"I typically use devices or technology when it is easy to set up, requires less steps, time or burden of down time for my patients or burden of time outside of my clinical treatment and day to day tasks"	"Using … 1-h of physical therapy and taking at least 20 min … to set up 4–6 electrodes on each leg before testing and trialing for one 30 min training session is not ideal and not supportive of her … goals and time frame for her … stay” (relative advantage)
	Adaptability	The degree to which an intervention can be adapted, tailored, refined, or reinvented to meet local needs	3/5 (60%) 4/9 (44.4%)	7 (4.0%)	“I can modify the speed …, body weight support, … [and] aspects of the set up … for those requiring a significant amount of assistance…” (design quality and packaging)	"Technology does not fit well in a small galley kitchen or in a tight spaced bathroom where my patient needed to practice"
	Design quality and packaging	Perceived excellence in how the intervention is bundled, presented, and assembled	2/5 (40%) 2/9 (22.2%)	6 (3.4%)	“…it is easy to operate with its portable remote to allow me to change parameters in real time and not have to stop if I am changing speed or inclines. It has built in safety/back up options if the portable remote did not work, but also for emergency stops, which also are adjustable …”	“In design, … It is not user friendly. As clinicians, we believe in being objective when we record treatment. Due to no easy, clear, objective ability to measure weight bearing … we cannot objectively document how much weight we are using ….”
	Relative advantage	Stakeholders’ perception of the advantage of implementing the intervention versus an alternative solution	5/5 (100%) 9/9 (100%)	32 (18.4%)	"…and if not for technology, [patients] would not be able to otherwise get up and perform this specific treatment."	"It is easier to setup a treatment using task-specific training with functional, everyday objects than try to make an unfamiliar device work." (complexity)
Outer setting	Clinician knowledge and beliefs about the intervention	Clinician's attitudes toward and value placed on the intervention as well as familiarity with facts, truths, and principles related to the intervention	5/5 (100%) 7/9 (77.8%)	18 (10.3%)	“It’s always easier to try new/unfamiliar interventions with patients who you have built a rapport with over time, and who you know would benefit from trying something new” (patient needs/resources)	"…we were hesitant to use it during a normal treatment time with patients since the device involved games when most of our patient's goals revolved around function (dressing, handwriting, etc.)."
	Clinician stage of change—readiness	Characterization of the phase a clinician is in, as he or she progresses toward skilled, enthusiastic, and sustained use of the intervention	4/5 (80%) 5/9 (55.6%)	10 (5.7%)	"I was lucky enough to learn how to set up a patient for [the device] while I was a student…" (clinician knowledge and beliefs)	"The device lived on our floor for a while, a daily reminder of the guilt of never finding time to improve familiarity."
	Other personal attributes of the clinician	A broad construct to include other personal traits such as tolerance of ambiguity, intellectual ability, motivation, values, competence, capacity, and learning style	4/5 (80%) 4/9 (44.4%)	4 (2.3%)	“After …residency early in my career, … I would consider myself an early adopter and enthusiast to learn new approaches for best patient outcomes…” (clinician knowledge and beliefs)	Despite being involved with technology in all roles, I would describe myself as a skeptic when it comes to consistent use of technology to treat the arm post neurologic injury
	Patient knowledge and beliefs about the intervention	Patient’s attitudes toward and value placed on the intervention as well as familiarity with facts, truths, and principles related to the intervention	2/5 (40%) 3/9 (33.3%)	4 (2.3%)	“He ended up loving this intervention, because to him it was the closest he was able to get to lifting weights (something that was really important to him prior to his injury.” (relative advantage)	“I also find that when meeting a patient for the first time, doing something that they can understand will help them get back home is pretty helpful in gaining rapport”
	Other personal attributes of the patient	A broad construct to include other personal traits such as tolerance of ambiguity, intellectual ability, motivation, values, competence, capacity, and learning style	5/5 (100%) 9/9 (100%)	27 (15.5%)	“Below is an example of a patient who checks all the boxes as being a “good candidate” for technology use- young, motivated, and willing to try anything.”	“However, I did not have many patients who would be appropriate for this equipment because they have cognitive deficits that limit attention, initiation, or comprehension of such games. Or they had extremely limited movement on their affected side.”
Inner setting (organization)	Patient needs and resources	The extent to which patient needs, as well as barriers and facilitators to meet those needs, are accurately known and prioritized by the organization	4/5 (80%) 6/9 (66.7%)	14 (8.0%)	"When it was time for him to leave, I provided him with information to get a cycle for home through the vendor that we had worked with, as she mentioned that she may be able to get him a cycle for a discounted rate since he was in the military."	"Finally, the patient’s wife is present and is very anxious, providing too many cues to the patient, overwhelming him and expressing disappointment with his performance."
	External policy	A broad construct that includes external strategies to spread interventions, including policy and regulations (governmental or other central entity), external mandates, recommendations and guidelines, pay-for-performance, collaboratives, and public or benchmark reporting	3/5 (60%) 3/9 (33.3%)	5 (2.9%)	[none coded]	"…how do I balance what is required vs what is ideal (required insurance tasks, progress updates, outcome measures, what has the patient already done for the day, what is recommended best available evidence for the interventions that address their goals)." (relative advantage, intervention evidence, patient attributes)
	Readiness to implement	Tangible and immediate indicators of organizational commitment to its decision to implement an intervention	4/5 (80%) 5/9 (55.6%)	12 (6.9%)	"[the vendor] was able to come to a co-treatment with the patient and I to help me with optimal setup."	"It had been a while since the 2-h training session that I went to …”
	Overall implementation climate	The absorptive capacity for change, shared receptivity of involved individuals to an intervention, and the extent to which use of that intervention will be rewarded, supported, and expected within their organization	5/5 (100%) 5/9 (55.6%)	6 (3.4%)	“…is integrated as part of the new hiring system …. In my experience, learning to correctly use the machine has not been any issues after initial training/mentoring.” (readiness to implement)	“My colleague and I both lacked confidence and did not want to use normal therapy time to test out the device, but we decided to treat patient pro bono after clinical hours to setup and run through with the device to improve familiarity.” (external policy, clinician attributes, clinician stage of change)
	Culture	Norms, values, and basic assumptions of a given organization	2/5 (40%) 2/9 (22.2%)	3 (1.7%)	“… appeal for “sexy” technology …”	"As an OT, we are taught 'function, function, function!', and this sentiment is reinforced by our professional organizations, and therapy leaders, that patient goals, daily interventions and treatment plans should address improved function."
Process of implementation	Executing	Carrying out or accomplishing the implementation according to plan	2/5 (40%) 2/9 (22.2%)	4 (2.3%)	"…we were assured that although the initial use would be confusing, consecutive uses become efficient."	"My confederate therapist and I could not coordinate another time to practice on the new device…"

*See https://cfirguide.org/constructs/ for full definitions of each code

**Where applicable, quotes that were double coded are noted in parentheses

Three reviewers coded each vignette in their entirety, but the vignettes are presented in a summarized form to follow the template more concisely and provide novel information. The full, unedited vignettes are available upon request. Summative content analysis included used the total number of codes presented, and the proportion of times each code was used across clinicians and vignettes [17]. This qualitative analysis plan provided a systematic method to synthesize the vignette results.

## Results

The constructs, their definitions, and results of summative content analysis are presented in Table 1.

Nine vignettes provided by five therapists detail experiences with patients with the following diagnoses: traumatic brain injury (n = 2), SCI (n = 1), stroke (n = 4), and multiple sclerosis (n = 1). Six vignettes were provided by OTs. Three vignettes were provided by PTs. All therapists have at least 4 years of clinical experience and have assisted with research projects in the past. The 17 codes (listed in Table 1) were applied 174 times. Most statements were coded with one code (n = 91), but when all three coders agreed, other statements were either double (n = 31), triple (n = 6) or quadruple (n = 3) coded. Each code is presented with exemplar quotes in Table 1, providing examples of the barriers and facilitators to implementation.

Content analysis of the 17 codes resulted in all five clinicians making statements coded as relative advantage, personal attributes of the patients, clinician knowledge and beliefs of the device/intervention, complexity of the devices, and overall implementation climate. Furthermore, all nine vignettes included statements coded as complexity, relative advantage, and personal attributes of the patient.

### Occupational therapy vignette 1

I would describe myself as a skeptic when it comes to consistent use of technology to treat the arm post neurologic injury. As an OT, we are taught “function, function, function!”, and this sentiment is reinforced by our professional organizations and therapy leaders. Patient goals and function inform treatment plans as paid for by Medicare/Medicaid, and treatment must improve functional Quality Reporting Measures (i.e., the ability to complete toilet transfers, dressing, grooming, etc.). It is challenging to connect devices with functional improvement, so I sparingly choose technology.

The patient was a 56-year-old male with left-sided (dominant) weakness after stroke. He had little distal active movement, was unable to participate in electrical stimulation because of the presence of a cardiac event monitor. His goals were feeding, reaching to cabinets, and managing medications, and he wanted to return to living alone after discharge. He was a “good candidate” for technology because young, motivated, and willing to try. However, the goal areas and need to be independent upon discharge swayed my decisions to use a mirror therapy protocol. The patient quickly progressed within 2 weeks to participate in a high repetition training. At this point technology such as an exoskeleton or smart glove could have been selected, but patient goals pushed me towards functional repetitive practice. We used forks, spoons, cups, coins, and standing to reach in our therapy kitchen and bathroom, areas where technology does not fit well. I hesitated to use technology because it could not have the patient working within 3 min of the start of the OT session, nor could it go home with him.

### Occupational therapy vignette 2

The hospital acquired a high-tech upper extremity exoskeleton that provided proximal support while a patient plays games on a screen. The hospital provided a half-day course for training; we were instructed that although the initial use would be confusing, consecutive uses become efficient. My colleague and I both lacked confidence and did not want to use normal therapy time to test out the device, but we decided to treat a patient pro bono after clinical hours to setup and run through with the device to improve familiarity.

The patient was a 50-year-old male who recently suffered a stroke resulting in left hemiparesis. We asked this patient to help primarily because he was able to verbally consent to extra treatment and had some movement in his left arm, which is rare in acute stroke. During the treatment session, we noticed that when the patient fatigued his flexor tone became worse, requiring removal of his arm from the exoskeleton for stretching. We did three rounds of 1–2 min of exercises, followed by a stretching break. This process repeated, resulting in a total of 9 min of treatment in a 40-min session, including setup. Then, the patient informed us of the need for the restroom, resulting in us ending the session earlier than anticipated.

My colleague and I could not coordinate another time to practice on the new device, and we were hesitant to use it during a normal treatment time with patients since the device involved games when most of our patient's goals revolved around function (dressing, grooming, etc.). It is easier to setup a treatment using task-specific training with functional, everyday objects than try to make an unfamiliar device work. The device lived on our floor for a while, a daily reminder of the guilt of never finding time to improve familiarity. However, I did not have many patients who would be appropriate for this equipment because they have physical deficits with extremely limited movement on their affected side or they have cognitive deficits that limit attention, initiation, or comprehension of such games. The device was eventually removed from the floor since it was unused by the therapy team.

### Occupational therapy vignette 3

A 75-year-old male with a prior medical history of atrial fibrillation, aortic valve repair, hypertension, epilepsy, and a prolonged (4 months) stay in a long-term acute care facility. He then had a subdural hematoma near the right frontal lobe resulting in a traumatic brain injury (TBI). His hospitalization was complicated by pneumonia, seizures, acute ischemic infarcts, and a tracheostomy placement. Impairments included bilateral upper extremity weakness (Action Research Arm Test: 0 left, 10 right), intention tremors in the right arm, decreased trunk control, decreased cervical control, and general malaise. Patient was unable to stand, walk, and hold himself upright in his wheelchair. He identified goals of “walking” and becoming more independent with brushing his teeth and getting dressed.

This was a 1-h session with a focus on grooming (oral hygiene and shaving) to improve arm control with both his upper extremities, strength, and upright head control. Since the patient had a low ARAT score on his left arm compared to his right, I decided to use a mobile arm support (MAS) to assist the patient with unweighting his left arm to engage bilaterally in the task. Setup took about 15 min, which involved retrieving the MAS from the gym, setting it up in the patient’s bathroom, wheeling the patient to the bathroom, and padding the device with towels to prevent his arm from slipping out. The patient had a lot of difficulty with incorporating his left arm into the task since he did not have effective grip strength, and overall, he required > 50% assistance with brushing his teeth due to difficulties reaching his face. The forward tilted wheelchair frequently led to his chin resting on his chest requiring the right arm to be propped on the sink. We then focused on task-oriented mass practice (retrieving toothbrush from the sink, moving it to the face, then placing it on a nearby table) assisted by the MAS, accomplishing only 15 repetitions because of frequent stops to readjust posture and the tension of the MAS system. This was difficult as I was simultaneously preventing falls out of the wheelchair, readjusting the MAS, cueing, and answering questions from the patient’s wife. The wife cued the patient but also expressed disappointment, which overwhelmed the patient. This took 35 min after the setup, with another 15 min for cleaning, moving the patient back, educating, and answering questions. Running 5 min late I excused myself, offering to check in again at the end of the day.

### Occupational therapy vignette 4

A young female in her twenties with no prior medical history presented 2 months after TBI resulting in multiple brain hemorrhages, diffuse axonal injury of the corpus callosum, splenium, and midbrain. She presented in a minimally conscious state (Rancho Los Amigos 3). She was non-verbal, restless, and moved her arm and right leg around non-purposefully with limited movement on her right side. She stared in the direction of sound cues most times and would follow some commands, but responses were inconsistent, delayed, and fleeting.

For this treatment, there were three areas of focus: functional communication, functional object use, and command follow. The communication device used in the session was a large button the patient was asked to hit with either her hand or foot. The patient successfully hit the device with her left foot in 4/20 trials. She also fatigued quickly from the activity, only able to try about 30 s at a time. She was unable to hit the device using her arm, and she repeatedly rubs her face and hair when cued to hit the target requiring hand-over-hand assistance. This process took about 25 min. We next tried brushing teeth with the left hand with cuing. The patient repeatedly brought the toothbrush to her mouth and chewed it in 3 instances after 20 min of engagement in the activity. During this time, the attending physician and students entered to watch and speak to the mother and the patient becomes fatigued and falls asleep in her wheelchair. I leave the session 5 min early.

### Occupational therapy vignette 5

As a float occupational therapist who sees patients on all different units, I am often seeing new patients each day that I have never met. I am a techno-phobe in my treatments because I feel strongly that using familiar objects/tasks are more motivating and patients understand how the intervention will be helpful to reach their goals. Consistent use of technology can be difficult because chart reviews may not paint the picture of how the patient is going to look when you finally see them.

A recent patient who suffered a stroke had visual deficits, left hemiparesis, was motivated, and wanted to work on visual scanning. I considered an interactive light board designed to train visual scanning and reaction timing, but it had been a while since the 2-h training session. When I am seeing patients more consistently and have built a rapport, I will know who will benefit from something new and I will brush up on my skills with such equipment. In this case, I chose to stick to familiar functional tasks and use time wisely, opting to use a visual scanning kit to organize a tackle box as shown in a picture. The patient had some difficulty completing it and needed some cueing, however, ultimately was able to complete the task with some support.

### Occupational therapy vignette 6

As an occupational therapist working with the Spinal Cord Injury population, I am always looking for ways to adapt every self-care task to allow them to be more independent. I worked with a young patient who was in a motor vehicle accident and sustained a C2 fracture, was on a ventilator, and had no movement in his upper or lower extremities other than the ability to shrug his shoulders. He was 19 years old and was in the US Army at the time of his accident. He did not have any surgical intervention, and when he was admitted he was classified as an ASIA B (sensory function preserved, motor function is not, below the level of injury).

Since he was active prior to injury, I thought he might enjoy the functional electrical stimulation (FES) bike as he became more stable. However, I was not as comfortable with setting up this piece of equipment at the time, so I contacted the vendor who is also an OT. The vendor attended, co-treated, and helped me with optimal setup. It was also helpful to have a second person since we added scapula and arm electrodes that took a long time. The patient loved this intervention because it was the closest to lifting weights, an activity that was important to him. Also, electrical stimulation below his injury level can see if he might regain strength in those muscles. When it was time for him to discharge, I provided him with information to get a cycle for home at a discounted military rate. This is one of my favorite interventions with SCI patients because of high repetitions and endurance, although it can take a while to set up.

### Physical therapy vignette 1

I am a PT working almost exclusively with traumatic and acquired brain injuries in an acute inpatient setting, and I am neutral to technology. There have been times when I frivolously support it, times I am skeptic, and times, I believe some devices will gather dust. The determining factor for my technology use is its adaptability, and if it’s quick to learn (~ 2 practice trials under supervision after formal training session). One of the pieces of technology I unequivocally support is Body Weight Supported Treadmill Training (BWSTMT). BWSTMT allows for earlier ambulation interventions, while assisting patient needs and high-intensity when appropriate. Speed can be modified to focus on aspects of gait that need more fine-tune control, such as step length or terminal knee extension. Other adaptations can be made to encourage upright trunk through use of strapping, additional inclines and allowance for backwards and lateral stepping. If not for such technology, I would be unable to perform this treatment. This also frees my hands, so I can assist with a specific target area (by positioning limbs in a specific phase in gaitor adding bolsters for a patient to step over) or stand back and see the bigger picture of gait mechanics. The portable remote allows me to change parameters such as speed and incline in real time, making the treadmill efficient to operate. Additionally, there are built-in safety/backup measures available and can be adjusted by the treating clinician.

As a student, I was lucky to work in a hospital that taught me how to set up and frequently use BWSTMT. Now as a Clinical Instructor for PT students, performing BWSTMT is an integral part of my daily practice. Thus, most students learn this as a part of their clinical experiences. For those who are unfamiliar with the BWSTMT, it takes practice, with variable patient assist levels, before using it independently. Often, practice is needed to learn more challenging set ups of the harness system on someone who requires 100% assist to don/doff a treadmill harness from a seated position and without a second hand to make the set-up smoother. Any sort of learning needed for this machine is integrated as part of the new hiring system.

While I am in full support of using BWSTMT for ambulation interventions, I have identified some drawbacks both in design and in application. Limitations in documenting body weight supported objectively or fitting the equipment to all patient sizes and shapes do not outweigh the positives. However, scheduling problems and patient cognition do limit my ability to use BWSTMT in a session. It can take about 15 min to set up someone and about 5 min to remove/clean up (less if the patient is higher level and the clinician is experienced and/or there is an extra assistor like a tech/aide). This amounts 20 min of therapy time. If a patient is scheduled for a 30-min session, or has an incontinent episode, a delay in medication, eating, or in bed at the beginning of therapy, it may be more effective and efficient to choose another treatment. Additionally, cognitive characteristics that accompany brain injury can decrease a patient’s tolerance for BWSTMT. A lack of insight into deficits in conjunction with a low frustration tolerance with high verbal and physical outbursts can impact safety of clinicians and patients, can reduce buy-in, or lead to treatment resistance. These limitations can present ethical implications. As a clinician, understanding how to modify the treatment parameters to the individual is just as important as knowing how to operate the technology itself.

### Physical therapy vignette 2

I am a PT with 9 years of experience in a large hospital, primarily inpatient. Because of my neurologic physical therapy residency, I am an early adopter, enthusiast, and proponent of novel technology, and have even participated in some efficacy and feasibility trials. My organization has many advanced devices available, but this does not equate to regular use unless it is easy to set up, does not cause patient downtime, or extensive personal time. Limits to technology use are: (1) is the device unready; (2) the patient is unready for treatment; (3) the patient goals do not match; (4) imbalance between organization or insurance requirements and ideal (evidence-based practice).

The patient was a 70-year-old female with right sided hemiparesis due to a left pare median pontine perforator infarct. Her goals were to improve walking speed and distance without assistance at home. I had a 60-min session and recognized the need for intense task-specific training. In previous sessions, she had difficulty reaching target heart rate due to poor right foot clearance, but more recently demonstrated improvement in proximal leg strength with remaining difficulty with knee control and foot clearance.

I chose to use BWSTMT to strengthen proximal leg muscles because we had equipment ready and I am extensively trained. To improve foot clearance, I had difficulty deciding whether to use a traditional ankle foot orthosis (AFO) or an FES orthosis. Although trained in FES, the barriers were (1) the device was four floors away; (2) the device was locked in a manager’s office because it is expensive; (3) I am required to send an email to the manager to check it out; (4) the device may not be charged; (5) I was unsure of where the electrodes were, and (6) if I delegate to a rehab aide, they might grab the incorrect one. Instead, I chose traditional AFO in the nearby cabinet, which worked okay but required more verbal cues more hands-on assistance for timing of step initiation and step lengths to maximize intensity.

The next day, I considered finding 20 min of personal time to locate and setup the FES device, but had an insurance progress note due, which meant I needed to assess the patient’s mobility skills and had limited time for gait training. The progress notes also led to extra documentation requirements, limiting my available time during lunch to track down the device. Additionally, I have some concerns with using this device in inpatient rehabilitation, for fear that it may not be covered by the patient’s insurance when they are discharged.

### Physical therapy vignette 3

A 48-year-old female with secondary progressive MS had goals to stand and make stepping actions. She had severe lower extremity weakness, already wore custom AFOs, and wanted to get stronger. Her parents were aging and were dependently lifting her in and out of bed. At the time, she required maximum assistance to stand, and we only had her for a short length of stay to get her home safely with less assistance since she already had good support at home. I considered the FES bike at one point but ultimately determined it would not have been beneficial time investment during inpatient rehabilitation as she was also seeing 1 h of speech therapy, 1 h of occupational therapy, and 1 h of physical therapy 5 of 7 days a week. Using her 1-h of physical therapy and taking at least 20 min or more to set up 4–6 electrodes on each leg before testing and trialing for one 30 min training session is not ideal for the time frame of her length of stay, and not supportive of her over all goals of transferring independently. Also, using the FES bike one time would not achieve the recommended frequency and duration to obtain the known benefits of the device. I chose to focus on slide-board transfers to reduce burden of care on her aging parents. Ultimately, her transfers improved significantly, and she was able to transfer herself using the slide-board.

## Discussion

Applying therapeutic RT for individuals with neurological impairments requires successful progression through a long and fragile chain of events: development, testing, validation, clinician uptake, and patient acceptance. Although each step presents its own set of challenges, we focused on the often overlooked but critical step of *clinician uptake* [12]. This study moved beyond surveys or focus groups to a vignette methodology that allowed us to understand clinicians’ real-time decision-making process in the moments when they are with their patients. We found that the five most common themes fell within the CFIR constructs of relative advantage, personal attributes of the patients, clinician knowledge/beliefs, device complexity (including time and setup), and organizational readiness to implement.

### Relative advantage

The most discussed barrier to using RT was its perceived relative disadvantage due to lack of relevance to everyday functional activities. Priority in this setting was task-specific practice of day-to-day functional activities. In contrast, many therapeutic RT enable repetitive practice, but usually in the form of games and simple strength and range of motion impairment-focused activities. Therapy outcomes are evaluated and reimbursed based on functional outcomes (e.g. bed mobility, transfers, walking, and self-care skills such as dressing, toileting, eating, and bathing) [18], rather than impairments or assessment scores (e.g. Action Research Arm Test, Fugl Meyer Assessment of Motor Recovery, Berg Balance Scale). Research has shown the biggest predictor of intention to use a RT is performance expectancy, or the degree to which an individual believes in the potential benefit of that RT [2, 19]. Thus, if therapists are unable to connect the impairment-focused task or game to a functional benefit they are less likely to use the device. RT endorsed in our vignettes increased repetitions of functional tasks and enabled patients to complete an action they could not otherwise complete, such as BWSTM and MAS systems. One possible solution to improve clinician uptake in inpatient rehabilitation, is for RT development to demonstrate the efficacy of RT in addressing functional outcomes. Furthermore, it may be of interest to developers to include functional metrics used by payer sources in the validation process and demonstration of efficacy of their devices.

### Patient attributes

Attributes of the patients and adaptability of the device determine clinician uptake and use of RT. Understanding the attributes of patients, as well as their needs and resources were common themes in the vignettes. In other studies, patient acceptance was a highly important factor for RT adoption [10]. Patient diagnoses, goals, and physical and cognitive abilities play a large role in guiding treatment decisions. Many engineering development studies exclude patients with cognitive deficits and can have very tight inclusion criteria related to physical function and sensation. Although necessary in the development phase, these restrictions limit generalizability in inpatient rehabilitation. It has been proposed that developers should clearly identify the appropriate patient population for their devices [4]. However, another solution that would be more valuable to clinicians is for developers to design quality devices that are adaptable to a variety of diagnoses, patient needs, and environments.

### Clinician knowledge and beliefs

A third CFIR construct endorsed by all five therapists was clinician knowledge and beliefs about the intervention. Clinicians’ individual experiences and comfort with RT, as well as their readiness to change, greatly influenced their decision to use RT in treatment. We found that clinicians appeared to value RT more when it is incorporated into their academic training, onboarding, or a part of their regular clinical practice. Providing only a single training session for complex RT may result in lower mastery or clinician self-efficacy, which are required for use with real patients. Interestingly, prior studies did not link RT uptake or barriers to therapists’ employment status, age, discipline, educational level, experience, or technology acceptance [2, 10, 14]. This suggests that clinician experience and training with specific RT may dictate RT uptake more than general clinician experience. One solution to improve clinician knowledge and beliefs about the intervention would be increase training duration to improve mastery or to incorporate technology training in schools.

### Device complexity and time

One of the most striking issues described narratively in the vignettes was that therapists have extraordinarily little time to use complex RT. Studies frequently mention the importance of simplicity, ease of set up, and convenient availability of RT [10, 14]. One limitation of the CFIR framework is that it does not include a specific construct for time. Instead, comments related to time barriers fell under several other constructs, including complexity, relative advantage, clinician stage of change, clinician knowledge and beliefs, implementation climate, and external policy. RT developers cannot create more time for therapist, and it is difficult for them to influence implementation climate and external policy. However, RT developers can create devices that are quick, intuitive, present a clear advantage over traditional interventions, and optimize clinical workflow. Additionally, training protocols should target moving the clinician through readiness to change into adoption of novel RT by addressing their knowledge and beliefs about the benefits of RT to support the amount of time it takes to use RT, particularly if the RT is perceived as complex.

### Organizational readiness to implement

Although RT developers may have low influence on organizational readiness, our vignettes support the importance of the organizational implementation climate for clinician uptake of RT. Implementation climate is defined as the “absorptive capacity for change, shared receptivity of involved individuals to an intervention, and the extent to which use of that intervention will be rewarded, supported, and expected within their organization” [15]. Further organizational investments in the implementation process are helpful to improve clinician uptake of devices. Examples of investments include time for practice and reflection, organizational incentives to increase motivation to use RT, as well as assistance with RT setup from rehabilitation technicians. Organizational implementation factors in the literature include support from the institution to facilitate use [2] and making RT use mandatory and seamless in clinical treatment [4]. Despite a goal of RT to supplement and assist therapists with treatment, it can place an unintentional burden on therapists when it comes to uptake and use in the clinic. Adding expectations for therapists to be trained on different RT oftentimes is an investment from therapist’s personal time, which in turn might contribute to burnout [12]. RT developers should consider how their devices fit into the overall organizational priorities and workflow to address the clinician uptake barrier related to organizational readiness to implement.

### Other constructs

Our interdisciplinary research team was surprised with infrequent mentions of evidence strength and cost compared to previously reported barriers to device implementation. Others have reported that clinicians rated cost as a very important acquisition factor [10, 14] or indicate cost as a barrier due to limited cost-effectiveness compared to intensive therapy evidence to warrant use [4, 20]. Our vignettes mentioned cost in the context of inconvenient procedural controls put in place to protect an expensive device or costs relative to a patient’s ability to acquire the device after discharge through insurance or other discounts. Other studies have shown that OTs and PTs rate evidence as the most important factor behind device use [14]. These vignettes dichotomously presented evidence strength as both allowing therapists to administer the number of repetitions recommended for significant clinical change; but also deterred another therapist due to inability to provide repeated use of RT to reach clinically significant gains due to short patient length of stay. The clinic already acquired and made the RT available to the clinicians in the vignettes, which suggests that the RT available to the therapists had enough evidence and were believed to be cost-effective to support the organization acquiring the device. These constructs suggest the importance of studying clinician uptake of RT after addressing the initial development and acquisition barriers.

## Limitations and directions for future research

Limitations are present in this analysis related to generalizability and the qualitative research methods. We acquired vignettes from one inpatient rehabilitation hospital with state-of-the-art RT, which may hold entrenched biases that are not reflective of the wider community of practitioners. A larger qualitative study exploring the barriers and facilitators in multiple organizations and therapists working in different rehabilitation setting would improve the generalizability. Additionally, we only look at the clinician perspective of RT uptake, when a more comprehensive approach would look at the perspectives of the patient and institution via administrators and management team. Technology use is a co-decision between the therapist and patient, and developers should consider both perspectives. Our report here should only be understood as a single snapshot of beliefs of rehabilitation therapists that still may be important for developers to understand as they prepare for the design or development process in this field. Related to the qualitative methods, our vignette prompt may have led to increased mentions of certain topics, such as the time taken to setup. A different vignette prompt could explore greater consideration of impairment-focused treatment and measurement RT. Additionally, we limited our coding to the 17 CFIR constructs that were already present in the literature, rather than inductive analysis to allow themes to emerge. However, using established implementation frameworks have demonstrated generalizability of findings in the presence of limited number of settings [21].

Future research and development should attend to determinants of successful clinical uptake supported in these vignettes. New RT should address relative advantage of functional task practice over impairment-focused interventions, the adaptability required to address varied patient populations, and the complexity of the RT. Developers also need to consider the importance of clinicians’ knowledge and beliefs about the intervention and support from the institution to facilitate a positive implementation climate.

## Conclusion

Considering clinician experiences will help developers to understand the complex clinical decision-making processes of the end users. The implementation science framework identifies actionable areas to improve RT development related to the intervention itself (advantages compared to traditional techniques and simplicity of design), the people involved in the intervention (attributes of patients, clinician knowledge/beliefs), as well as the organizations’ implementation climate. Future research addressing these areas can aide in development and clinical integration of innovative RT.

## Data Availability

The datasets generated during and/or analyzed during the current study are available from the corresponding author on reasonable request.
